# *IL5* rs2069812 and *IL13* rs1800925 Genetic variants as key determinants of clinically relevant asthma phenotypes

**DOI:** 10.1371/journal.pone.0354597

**Published:** 2026-07-24

**Authors:** Pattara Kanoksing, Apichaya Puangpetch, Theerasuk Kawamatawong, Thatchathum Kuttiyod, Kantapat Simmalee, Roger Frutos, Putthapoom Lumjiaktase

**Affiliations:** 1 Department of Pathology, Faculty of Medicine Ramathibodi Hospital, Mahidol University, Bangkok, Thailand; 2 Division of Pulmonary and Critical Care Medicine, Department of Medicine, Faculty of Medicine Ramathibodi Hospital, Mahidol University, Bangkok, Thailand; 3 Ratchasuda Institute, Faculty of Medicine Ramathibodi Hospital, Mahidol University, Bangkok, Thailand; 4 Cirad, Intertryp U.M.R.17, Montpellier, France; 5 Department of Health, Faculty of Vocational Studies, Universitas Airlangga, Surabaya, Indonesia; UTRGV: The University of Texas Rio Grande Valley, UNITED STATES OF AMERICA

## Abstract

Type 2-high airway inflammation in adults with asthma is heavily driven by the cytokines interleukin (IL)-4, IL-5, and IL-13. While genetic variations in these cytokines are known to influence asthma pathogenesis, their specific impacts on clinically relevant phenotypes remain to be fully elucidated. This study aimed to investigate the associations between inflammatory cytokine gene polymorphisms, clinical asthma phenotypes, inflammation cell subtypes, and lung function**.** A cross-sectional study was conducted involving 125 adults with asthma. Genotyping was performed for the following single-nucleotide polymorphisms (SNPs): *IL33* (rs1342326, rs3939286), *IL4* (rs2243250, rs2243248), *IL5* rs2069812, and *IL13* (rs20541, rs1800925). Clinical evaluations included lung function, blood eosinophils, type 2 innate lymphoid cells (ILC2s), Th2 cells, cytokine levels, and specific IgE. The *IL4* rs2243248 TT genotype was associated with higher TNF-α (*p* = 0.038), while the *IL13* rs1800925 polymorphism was associated with increased Th2 cell counts (*p* = 0.025). Notably, *IL5* rs2069812 was strongly associated with blood eosinophilia (*p* < 0.001) and reduced lung function. Linear regression revealed a significant gene-dose effect of IL-5 rs2069812 T allele, which correlated with an increase in log_10_ eosinophils (*β* = 0.207, *p* < 0.001) and a decrease in post-bronchodilator FEV1% (*β* = −7.38, *p* = 0.014). Furthermore, the *IL5* rs2069812 and *IL13* rs1800925 variants significantly increased the risk of blood eosinophilia (Prevalence Ratio [PR] = 2.59, *p* < 0.001) and fixed airflow obstruction (PR = 1.81, *p* = 0.039), respectively. The *IL5* rs2069812 and *IL13* rs1800925 polymorphisms serve as key genetic determinants of persistent blood eosinophilia and fixed airflow obstruction, respectively. Both variants significantly contribute to the severity of airflow limitation in adult asthma, highlighting their potential as biomarkers for precision phenotyping.

## Introduction

Asthma is a heterogeneous chronic inflammatory airway disease affecting approximately 300 million people worldwide [1]. The disease is characterized by various clinical phenotypes driven by complex gene-environment interactions [2]. Among these, type 2-high airway inflammation is the most prevalent, affecting up to two-thirds of adult patients with asthma [3]. This inflammatory pathway is predominantly orchestrated by T helper 2 (Th2) cells, and increasingly recognized type 2 innate lymphoid cells (ILC2s), and is functionally subdivided into allergic and eosinophilic phenotypes. The cytokines interleukin (IL)-4, IL-5, and IL-13 act as central mediators in this cytokine-driven cascade, which is frequently exacerbated by environmental triggers such as allergens, viruses, and pollutants. Specifically, IL-4 and IL-13 are indispensable for driving allergic inflammation and airway remodeling, whereas IL-5 is the critical cytokine responsible for eosinophil maturation, activation, and survival [4].

Beyond environmental exposures, emerging evidence highlights that genetic variations, particularly single-nucleotide polymorphisms (SNPs) encoding these key inflammatory cytokines, significantly influence asthma susceptibility and severity. Notable genetic variants include promoter polymorphisms in *IL4* (rs2243248 C/T), *IL5* (rs2069812 C/T), and *IL13* (rs1800925 C/T), as well as untranslated region (UTR) variants in *IL4* (rs2243250 C/T) and *IL13* (rs20541 A/G). These variants have been associated with an increased risk of asthma across diverse demographic groups. For instance, the *IL4* rs2243250 polymorphism confers asthma risk across Asian, European, and American populations [5] and correlates with elevated total serum IgE levels in Japanese cohorts [6]. Similarly, the *IL4* rs2243248 variant increases the likelihood of developing asthma in Asian populations [7]. Furthermore, both *IL13* rs1800925 and rs20541 have been identified as susceptibility loci in Asian and Caucasian groups [8], rs1800925 linked to elevated IgE in children [9], and rs20541 associated with airway hyperresponsiveness in Japanese adults with asthma [10].

Despite the extensive identification of asthma-associated genetic variants, their precise functional impacts on clinically relevant immunological and physiological phenotypes remain underexplored. Previous genetic studies have predominantly focused on generalized asthma risk, often lacking comprehensive profiling of specific clinical outcomes such as inflammatory cell subtypes and progressive lung function decline in adult cohorts. Addressing this knowledge gap is essential for stratifying patients based on distinct molecular mechanisms and advancing precision medicine. Therefore, this study aims to comprehensively investigate the associations between key inflammatory cytokine gene polymorphisms (*IL4*, *IL5*, and *IL13*) and specific asthma phenotypes, encompassing cellular inflammation profiles, the development of fixed airflow obstruction (FAO), and objective clinical outcomes, including lung function parameters in adult patients with asthma.

## Materials and methods

### Study population

To investigate genotype-phenotype associations in asthma, a cross-sectional study was conducted using archived biological specimens from 125 Thai patients with asthma. Participants were originally recruited between 2 November 2022 and 1 November 2023 at the chest clinic of Ramathibodi Hospital. All patients were diagnosed according to the Global Initiative for Asthma (GINA) guidelines and were being treated with inhaled corticosteroids (ICS). Patients who experienced an exacerbation due to allergens, infection, or pollution within the past eight weeks or who had used systemic corticosteroid medication within the past eight weeks were excluded. Additionally, individuals receiving monoclonal antibody therapies targeting IL-5, IL-4, or IL-13, as well as those with a history of tobacco smoking of ≥10 pack-years, patients with parasitic infections, systemic autoimmune diseases, or hematologic malignancies, were excluded. The original collection of samples was approved by the Human Research Ethics Committee of the Faculty of Medicine Ramathibodi Hospital, Mahidol University (COA. MURA2022/631) on 2 November 2022. Written informed consent was obtained from all participants at the time of recruitment for the long-term storage and future genetic analysis of their samples. The present study represents a secondary analysis approved under COA. MURA2023/435 on 22 May 2023. Whole blood samples were collected using containing EDTA Vacutainer tubes and stored at −80°C for subsequent DNA extraction.

### Lung function assessment

Spirometry and bronchodilator reversibility tests were performed in accordance with the American Thoracic Society (ATS)/European Respiratory Society (ERS) guidelines to assess lung function by measuring forced expiratory volume in one second (FEV_1_) and forced vital capacity (FVC). For the lung function assessment, patients were categorized into specific phenotypes based on the presence of fixed airflow obstruction (FAO). FAO was determined using the post-bronchodilator FEV_1_ as a percentage of the predicted value (FEV_1_% predicted). Consequently, patients were divided into two groups: those with FAO (post-bronchodilator FEV_1_% predicted < 70%) and those without FAO (post-bronchodilator FEV_1_% predicted > 70%). Additionally, bronchodilator reversibility was evaluated following the administration of 400 µg of inhaled salbutamol. Patients were then classified into two groups: those with bronchodilator reversibility (an FEV_1_ increase of ≥ 12% and ≥ 200 mL from baseline) and those without reversibility (an FEV_1_ increase of < 12% or < 200 mL from baseline).

### DNA extraction and SNPs genotyping

Genomic DNA was extracted from 400 µL of EDTA-anticoagulated whole blood samples using the MagNA Pure automated extraction system (Roche Applied Science, Penzberg, Germany). This automated process involved cell disruption, protein digestion, magnetic bead-based nucleic acid binding, washing, and final elution. Potential single-nucleotide polymorphisms (SNPs) associated with interleukin genes and asthma susceptibility were selected utilizing the NCBI dbSNP database and previous literature. Specifically, seven variants were selected for genotyping: *IL4* rs2243250 [5], *IL4* rs2243248 [7], *IL5* rs2069812 [11], *IL13* rs20541 [10], *IL13* rs1800925 [9], *IL33* rs1342326, and *IL33* rs3939286 [12]. Genotyping was performed using a TaqMan real-time PCR assay (Applied Biosystems Inc., Carlsbad, CA, USA) in according with the manufacturer's instructions. All amplification and allelic discrimination experiments were conducted using the StepOne Real-Time PCR System instrument (Applied Biosystems Inc., Carlsbad, CA, USA).

### Measurement of Serum allergen sIgE

Serum sIgE levels were measured to assess sensitization to 18 aeroallergens (House dust mite, American cockroach, Timothy grass, Bahia grass, *Candida albicans*, *Penicillium chrysogenum*, Cat dander, Bermuda grass, Meadow grass, Acacia, *Alternaria alternata*, *Cladosporium herbarum*, Dog dander, Rye-grass, Johnson grass, Careless weed, *Setomelanomma rostrata*, and *Aspergillus fumigatus*). These measurements were performed using the ImmunoCap with Fluoro-Enzyme Immunoassay (FEIA) on an automated Phadia instrument (Phadia, Biomed Diagnostics, Bangkok, Thailand). Allergen sensitization was defined as an sIgE level ≥ 0.35 kUA/L, whereas a level < 0.35 kUA/L was considered negative.

### Measurement of cytokines and inflammatory cells

Both cytokine and inflammatory cell analyses were performed using a BD FACSLyric™ a flow cytometer (Becton Dickinson, Franklin Lakes, NJ, USA). Serum levels of IL-4, IL-5, IL-6, IL-10, IL-13, and TNF-α were quantified using the bead-based immunoassay **(**LEGENDplex™ Human Cytokine Panel 13-plex kit, Cat. No. 740726; BioLegend, San Diego, CA, USA) with a filter plate, according to the manufacturer’s instructions. For cellular analysis, Inflammatory cells were identified using whole blood stained with specific antibody cocktail for Th2 cells (APC-H7 Mouse Anti-Human CD45, Mouse Anti-Human CD3, FITC Mouse Anti-Human CD4 and CRTH22), eosinophils (PE Mouse Anti-Human CD45, BB515 Mouse Anti-Human CD66b, and PE Mouse Anti-Human CD16), Th cells (APC-H7 Mouse Anti-Human CD45, Mouse Anti-Human CD3 and FITC Mouse Anti-Human CD4), and ILC2s (APC-H7 Mouse Anti-Human CD45, PerCP Mouse Anti-Human CD3, BB515 Mouse Anti-Human CD4, BV510 Mouse Anti-Human CRTH2, and PE Mouse Anti-Human CD117). The mixtures were then gently vortexed and incubated in the dark at room temperature. Subsequently, 1X FACS Lysing Solution was added, and the mixtures were gently vortexed again, followed by an additional incubation in the dark at room temperature. The samples were thoroughly mixed immediately before analysis.

### Asthma control and severity assessment

Asthma control was evaluated using the Thai version of the Asthma Control Test (ACT), which assesses symptoms over the preceding four weeks. Participants were categorized into two groups following a previous study [13]: those with controlled asthma (ACT score > 19) and those with uncontrolled asthma (ACT score ≤ 19). In addition, asthma airflow limitation severity was assessed using the pre-bronchodilator FEV_1_ percentage of the predicted value. Patients were stratified into a mild asthma airflow limitation group (pre-bronchodilator FEV_1_% predicted > 70% predicted) and a moderate-to-severe asthma airflow limitation group (pre-bronchodilator FEV < 70% predicted) [14].

### Statistical analysis

SNPs were assessed for deviation from Hardy-Weinberg equilibrium (HWE) through chi-square (χ2) goodness-of-fit tests, with expected frequencies derived from allele frequencies. Categorical variables were compared using the χ2 test, whereas the Kruskal–Wallis test was employed to analyze non-normally distributed continuous data. Continuous variables exhibiting skewed distributions were log10-transformed prior to analysis. Linear regression models were used to evaluate the additive effect of genotypes on quantitative traits, with results reported as beta coefficients (*β*), standard errors (SE), and R-squared (*R*^2^) values. For the genotype-phenotype association analyses, patients with asthma were stratified into distinct phenotypic groups. In these comparisons, the subgroup with the less severe phenotype or the most common homozygous genotype served as the reference group for calculating the prevalence ratio (PR). Generally, a *p*-value < 0.05 was considered statistically significant. However, to account for multiple hypothesis testing, a Bonferroni correction was applied, establishing a stringent significance threshold of *p* < 1.25 × 10 ⁻ ⁴ for the primary association analyses. All statistical analyses were performed using Stata version 17 (StataCorp LLC, College Station, TX, USA) and GraphPad Prism version 9 (GraphPad Software, San Diego, CA, USA).

## Results

A total of 125 patients with asthma (86 females, 39 males) receiving ICS treatment were recruited from the chest clinic of Ramathibodi Hospital. The participants had a mean age of 64.63 ± 13.44 years. Clinical and immunological parameters, including serum sIgE levels, spirometry measurements (including percentage of predicted values), serum inflammatory cytokine concentrations, and circulating inflammatory cell counts, were comprehensively recorded. The demographic and clinical characteristics of the study cohort are summarized in Table 1.

**Table 1:** Demographic and biochemical profile of asthma patients.

Demographic Categories	Asthma patients (n = 125)
Gender (n, %)	
Female	86 (68.80%)
Male	39 (31.20%)
Age (year, mean±SD)	64.63 ± 13.44
Specific-IgE (kUA/L; median, min-max)	0.27 (0.02-101)
Spirometry (mean ±SD; median, min-max)	
Pre-BD FEV_1_ (%pred)	66.29 ± 23.03
Post-BD FEV_1_ (%pred)	67.56 ± 23.60
%ΔFEV_1_ (%)	6 (−7.69-79.49)
Inflammatory Cytokine Levels (pg/ml; median, min-max)	
Interleukin-4	0 (0.00-93.12)
Interleukin-5	0.27 (0-17.16)
Interleukin-6	0.1 (0-31.65)
Interleukin-10	0.03 (0-16.46)
Interleukin-13	0 (0-164.84)
TNF-α	0 (0-128.77)
Inflammatory Cell Counts (Cells/uL; mean ±SD; median, min-max)	
Blood eosinophil count	204 (10-1635)
Th cells	791.2 ± 375.7
ILC2 cells	109 (2-476)
Th2 cells	124 (0-1567)
% Th of Lymphocytes	36.8 ± 8.1
% ILC2 of Th cells	10.1 (1.2-64.6)
% Th2 of Th cells	14.7 (0-99.7)

Pre-BD: pre-bronchodilator, Post-BD: post-bronchodilator, FEV_1_: Forced expiratory volume in one second, FVC: Forced vital capacity.

The genotype frequencies of the seven single-nucleotide polymorphisms were assessed for deviation from Hardy-Weinberg equilibrium (HWE). Two variants within the IL33 gene (rs1342326 and rs3939286) deviated significantly (*p* < 0.001) from HWE and were consequently excluded from subsequent analyses. These data are provided in **S1 Table**. The genotype distributions of the remaining five SNPs were consistent with HWE (**Table 2**).

**Table 2:** Expected frequencies of genotypes of each single nucleotide polymorphism using the Hardy-Weinberg equilibrium.

Gene positions	Genotype	Observed asthma patients	Expected asthma patients	P-value
*IL4* (rs2243250 C/T)	CC	15	15.84	0.832
	CT	59	57.31	0.824
	TT	51	51.84	0.905
*IL4* (rs2243248 C/T)	CC	14	15.84	0.643
	CT	61	57.31	0.625
	TT	50	51.84	0.798
*IL13* (rs1800925 C/T)	CC	80	78.41	0.858
	CT	38	41.18	0.619
	TT	7	5.41	0.494
*IL13* (rs20541 A/G)	AA	20	21.22	0.791
	AG	63	60.56	0.754
	GG	42	43.22	0.853
*IL5* (rs2069812 C/T)	CC	60	57.12	0.703
	TC	49	54.75	0.437
	TT	16	13.12	0.426

### Comparison of clinical and immunological profiles according to interleukin genotypes in patients with asthma

Clinical and immunological profiles, including serum sIgE, circulating inflammatory cytokines, inflammatory cell counts (blood eosinophils, Th cells, ILC2s, and Th2 cells), and pre- and post-bronchodilator spirometric parameters were compared across the five evaluated single-nucleotide polymorphisms (SNPs): *IL4* (rs2243250 and rs2243248), *IL13* (rs20541 and rs1800925), and *IL5* (rs2069812). The Kruskal-Wallis test revealed significant phenotypic variations associated with specific genotypes.

**Fig 1:**
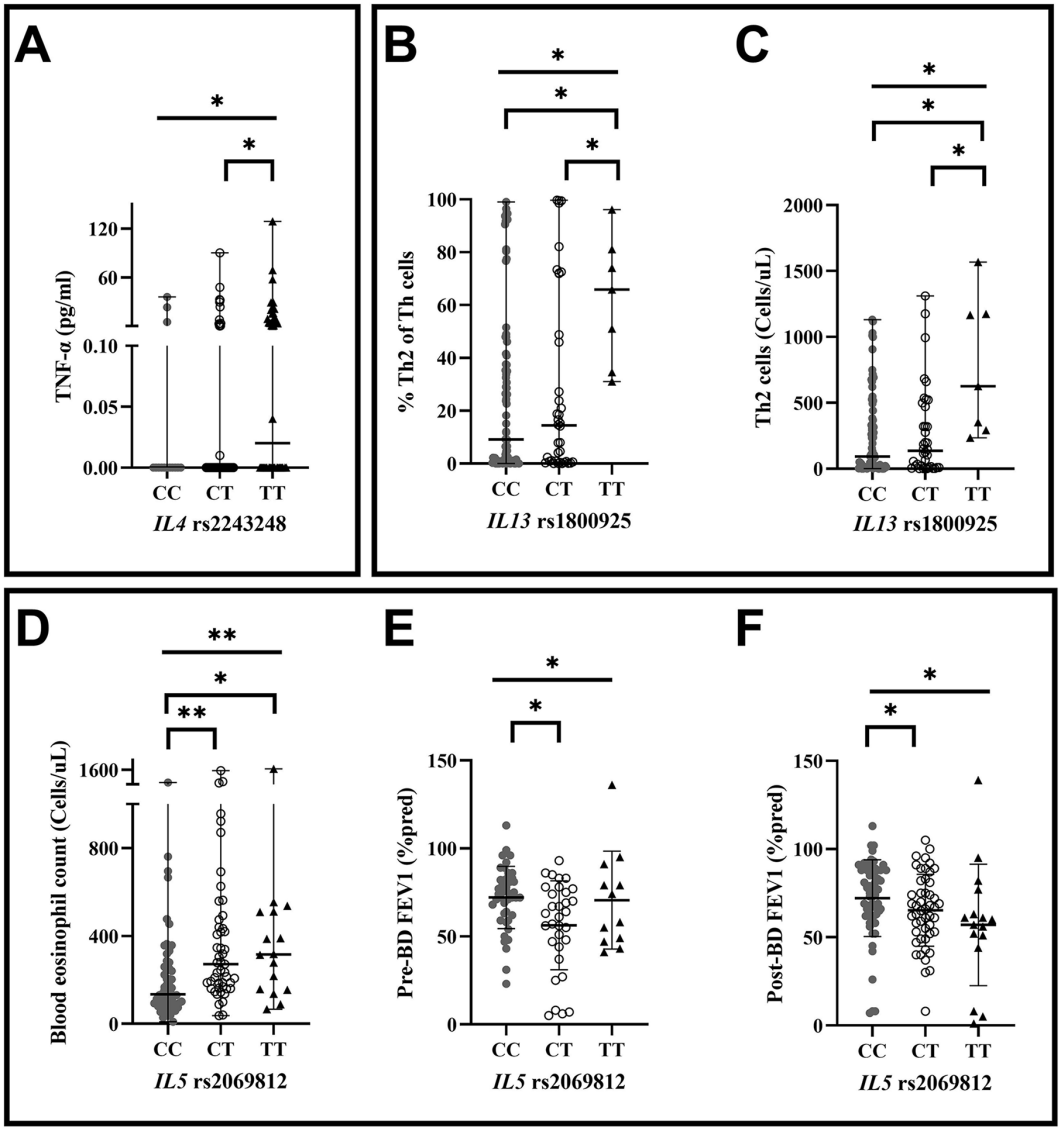
Comparison of clinical and immunological profiles according to interleukin genotypes. Clinical and immunological parameters are stratified by (A) *IL4* rs2243248, (B) *IL13* rs1800925, and (C) *IL5* rs2069812 genotypes, including homozygous wild type, heterozygous variant, and homozygous variant in asthma patients. Data are presented as medians with interquartile range (IQR). *Statistical significance difference was defined as *p* < 0.05. **Significance after Bonferroni correction (*p* < 1.25x10^-4^).

Regarding the *IL4* rs2243248 polymorphism, TNF-α levels were significantly elevated in individuals with the TT genotype compared to those with the CC (*p* = 0.0386) and CT (*p* = 0.0035) genotypes (Fig 1A). For *IL13* rs1800925, significant differences were observed across all genotype comparisons for both the percentage of Th2 cells among Th cells (TT vs. CC, *p* = 0.0286; TT vs. CT, *p* = 0.015; CT vs. CC, *p* = 0.0078) and total Th2 cell counts (TT vs. CC, *p* = 0.011; TT vs. CT, *p* = 0.005; CT vs. CC, *p* = 0.0024) (Fig 1B and 1C).

Notably, the *IL5* rs2069812 polymorphism strongly impacted both inflammatory and clinical parameters. Blood eosinophil counts were significantly higher in the TT and CT genotypes compared to the CC genotype (both *p* < 0.001), with the TT genotype also exhibiting significantly higher counts than the CT genotype (*p* = 0.001) (Fig 1D). Clinically, individuals with the CC genotype demonstrated significantly better lung function, showing higher pre- and post-bronchodilator FEV1% predicted values compared to the CT (pre-BD, *p* = 0.0078; post-BD, *p* = 0.0083) and TT genotypes (pre-BD, *p* = 0.033; post-BD, *p* = 0.012) (Fig 1E and 1F).

**Fig 2:**
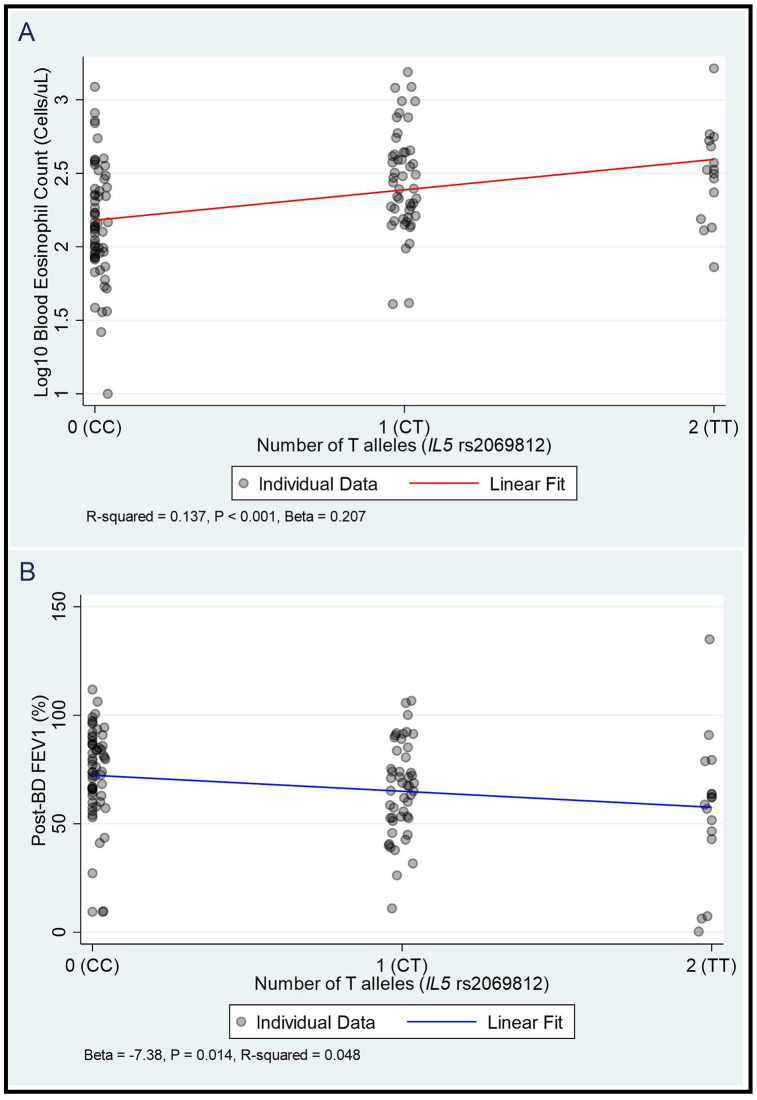
Linear regression analysis of the gene-dose effect of *IL5* rs2069812 on inflammatory and clinical markers in asthmatic patients. (A) Regression analysis demonstrated that each additional T allele of *IL5* rs2069812 is associated with a significant increase in log₁₀-transformed blood eosinophil counts (*β* = 0.207, *R*² = 0.137, *p* < 0.001). (B) Regression analysis showed a corresponding negative trend, with each additional T allele associated with a significant decrease in post-bronchodilator FEV₁% (*β* = −7.38, *R*² = 0.048, *p* = 0.014).

Linear regression analysis further confirmed a robust gene-dose effect for *IL5* rs2069812: each additional T allele was associated with a significant quantitative increase in log10-transformed eosinophil counts (*β* = 0.207, *p* < 0.001; Fig 2A) and a corresponding negative trend in post-bronchodilator FEV1% predicted values (*β* = −7.38, *p* = 0.014; Fig 2B).

In contrast, no significant phenotypic associations were observed for the *IL4* rs2243250 and *IL13* rs20541 variants. Furthermore, the remaining clinical and immunological parameters showed no significant differences when stratified by the *IL4* rs2243248, *IL13* rs1800925, and *IL5* rs2069812 genotypes. Comprehensive results for these analyses are detailed in S2 Table (for *IL4* rs2243248, *IL13* rs1800925, and *IL5* rs2069812) and S3 Table (for *IL4* rs2243250 and *IL13* rs20541).

### Associations of interleukin genotype with asthma phenotype factors in asthma

Patients were stratified into six distinct phenotypic groups for association analyses: (1) allergen sensitization (sIgE ≥ 0.35 vs. < 0.35 kUA/L); (2) blood eosinophilia (eosinophils ≥ 150 vs. < 150 cells/µL); (3) asthma airflow limitation severity (pre-bronchodilator FEV1 ≥ 70% [mild] vs. < 70% [moderate-to-severe] of predicted); (4) asthma control (ACT score ≥ 20 [controlled] vs. ≤ 19 [uncontrolled]); (5) bronchodilator reversibility (FEV1 increase ≥ 12% and ≥ 200 mL vs. < 12% or < 200 mL from baseline); and (6) fixed airflow obstruction (FAO; post-bronchodilator FEV1 < 70% vs. ≥ 70% of predicted). Associations between these phenotypes and interleukin genotypes were evaluated using the Chi-square test.

Regarding the *IL13* rs1800925 polymorphism, the T allele was associated with a higher prevalence of blood eosinophilia compared to the C allele (Prevalence Ratio [PR] = 1.81, *p* = 0.039). Additionally, the CT genotype conferred a higher risk of moderate-to-severe asthma compared to the CC genotype (PR = 2.03, *p* = 0.034) (Table 3).

**Table 3:** Associations of IL13 rs1800925 genotype with asthma phenotype in asthma patients.

*IL13* rs1800925 genotype								
	Genotypes	N	Allergen sensitization (n, %)		PR (95% CI)	*p*-value		
			sIgE ≥ 0.35 kUA/l	sIgE < 0.35 kUA/l				
	CC	80	38 (64.4)	42 (63.6)		0.386	Ref.	
	CT	38	16 (27.1)	22 (33.3)			0.582	Ref.
	TT	7	5 (8.4)	2 (3.0)			0.225	0.153
	T allele	52	26 (22.0)	26 (19.6)	1.12 (0.69-1.81)	0.649		
	C allele	198	92 (77.9)	106 (80.3)				
**Dominant pattern**	CT + TT	45	21 (35.5)	24 (36.3)	0.98 (0.61-1.56)	0.928		
	CC	80	38 (64.4)	42 (63.6)				
**Recessive pattern**	TT	7	5 (8.4)	2 (3.0)	2.79 (0.56-13.87)	0.186		
	CT + CC	118	54 (91.5)	64 (96.9)				
	**Genotypes**	**N**	**Blood eosinophilia (n, %)**		**PR (95% CI)**	***p*-value**		
			**EOS ≥ 150 Cells/uL**	**EOS < 150 Cells/uL**				
	CC	80	47 (58.0)	33 (75.0)		0.173	Ref.	
	CT	38	28 (34.5)	10 (22.7)			0.115	Ref.
	TT	7	6 (7.4)	1 (2.3)			0.161	0.496
	T allele	52	40 (24.7)	12 (13.6)	1.81 (1.00-3.27)	0.039*		
	C allele	198	122 (75.3)	76 (86.4)				
**Dominant pattern**	CT + TT	45	34 (41.9)	11 (25.0)	1.68 (0.95-2.97)	0.059		
	CC	80	47 (58.0)	33 (75.0)				
**Recessive pattern**	TT	7	6 (7.4)	1 (2.3)	3.26 (0.41-26.22)	0.233		
	CT + CC	118	75 (92.6)	43 (97.7)				
	**Genotypes**	**N**	**Asthma airflow limitation severity (n, %)**		**PR (95% CI)**	***p*-value**		
			**Pre-BD FEV**_**1**_ **< 70%**	**Pre-BD FEV**_**1**_ **≥ 70%**				
	CC	57	23 (54.7)	34 (73.9)		0.115	Ref.	
	CT	26	17 (40.4)	9 (19.5)	2.03 (1.02-4.02)		0.034*	Ref.
	TT	5	2 (4.7)	3 (6.5)			0.987	0.285
	T allele	36	21 (25.0)	15 (16.3)	1.53 (0.84-2.77)	0.153		
	C allele	140	63 (75.0)	77 (83.7)				
**Dominant pattern**	CT + TT	31	19 (45.2)	12 (26.1)	1.73 (0.96-3.13)	0.06		
	CC	57	23 (54.7)	34 (73.9)				
**Recessive pattern**	TT	5	2 (4.7)	3 (6.5)	0.73 (0.13-4.16)	0.721		
	CT + CC	83	40 (95.2)	43 (93.4)				
	**Genotypes**	**N**	**Asthma controlled (n, %)**		**PR (95% CI)**	***p*-value**		
			**ACT score≤19**	**ACT score>19**				
	CC	68	13 (68.4)	55 (61.7)		0.911	Ref.	
	CT	34	5 (26.3)	29 (32.5)			0.581	Ref.
	TT	6	1 (5.2)	5 (5.6)			0.883	0.901
	T allele	46	7 (18.4)	39 (21.9)	0.84 (0.41-1.73)	0.633		
	C allele	170	31 (81.5)	139 (78.0)				
**Dominant pattern**	CT + TT	40	6 (31.5)	34 (38.2)	0.82 (0.41-1.68)	0.587		
	CC	68	13 (68.4)	55 (61.8)				
**Recessive pattern**	TT	6	1 (5.2)	5 (5.6)	0.93 (0.11-7.56)	0.951		
	CT + CC	102	18 (94.7)	84 (94.3)				
	**Genotypes**	**N**	**Bronchodilator reversibility (n, %)**		**PR (95% CI)**	***p*-value**		
			**FEV**_**1**_ **increase of <12% and <200 mL from baseline**	**FEV**_**1**_ **increase of ≥12% and ≥200 mL from baseline**				
	CC	25	24 (29.62%)	1 (16.66%)		0.309	Ref.	
	CT	57	52 (64.19%)	5 (83.34%)			0.444	Ref.
	TT	5	5 (6.19%)	0 (0.00%)			0.649	0.489
	T allele	5	62 (38.27%)	5 (41.66%)	0.99 (0.91-1.07)	0.815		
	C allele	107	100 (61.73%)	7 (58.34%)				
**Dominant pattern**	CT + TT	62	57 (70.37%)	5 (83.34%)	0.96 (0.86-1.07)	0.498		
	CC	25	24 (29.63%)	1 (16.66%)				
**Recessive pattern**	TT	5	5 (6.17%)	0 (0.00%)	1.07 (1.01-1.14)	0.531		
	CT + CC	82	76 (93.83%)	6 (100.00%)				
	**Genotypes**	**N**	**Fixed airflow obstruction (n, %)**		**PR (95% CI)**	***p*-value**		
			**Post-BD FEV**_**1**_ **< 70%**	**Post-BD FEV1 ≥ 70%**				
	CC	80	38 (56.7)	42 (72.4)		0.168	Ref.	
	CT	38	24 (35.8)	14 (24.1)			0.111	Ref.
	TT	7	5 (7.4)	2 (3.4)			0.224	0.674
	T allele	52	34 (25.3)	18 (15.5)	1.63 (0.97-2.73)	0.055		
	C allele	198	100 (74.6)	98 (84.4)				
**Dominant pattern**	CT + TT	45	29 (43.2)	16 (27.5)	1.56 (0.95-2.58)	0.068		
	CC	80	38 (56.7)	42 (72.4)				
**Recessive pattern**	TT	7	5 (7.4)	2 (3.4)	2.16 (0.44-10.74)	0.33		
	CT + CC	118	62 (92.5)	56 (96.5)				

*Nominal significance (*p* < 0.05), **Significance after Bonferroni correction (*p* < 1.25x10^-4^). N: Number of patients, PR: Prevalence ratio, sIgE: Specific-IgE, EOS: Eosinophils, ACT: Asthma control test, FEV1: Forced expiratory volume in one second, Pre-BD FEV_1_: pre-bronchodilator FEV_1_, Post-BD FEV_1_: post-bronchodilator FEV_1._

For the *IL5* rs2069812 variant, the T allele was associated with an increased risk of allergen sensitization compared to the C allele (PR = 1.47, *p* = 0.035). Notably, this polymorphism demonstrated a profound association with blood eosinophilia across the CC, CT, and TT genotypes (*p* < 0.001), as well as under a dominant inheritance model (*p* < 0.001). Specifically, the CT and TT genotypes were associated with a 3.09- and 3.90-fold higher risk of blood eosinophilia than the CC genotype (*p* < 0.001 and *p* = 0.009, respectively). At the allelic level, the T allele increased the risk of blood eosinophilia by 2.59-fold compared to the C allele (*p* < 0.001). Crucially, the association between the *IL5* rs2069812 polymorphism and blood eosinophilia remained robustly significant even after applying the stringent Bonferroni correction (*p* = 3.47x10^‒5^).

Furthermore, significant differences were observed between the *IL5* rs2069812 genotypes and moderate-to-severe asthma (*p* = 0.023 overall; *p* = 0.010 for the dominant model). The CT genotype (PR = 2.12, *p* = 0.006) and the T allele (PR = 1.57, *p* = 0.042) were both associated with a higher risk of moderate-to-severe asthma compared to the CC genotype and C allele, respectively. Similarly, this variant was significantly associated with FAO (*p* = 0.023 overall; *p* = 0.010 for the dominant model). The CT and TT genotypes exhibited a 1.55- and 3.16-fold higher risk of FAO compared to the CC genotype (*p* = 0.042 and *p* = 0.017, respectively), while the T allele conferred a 1.73-fold higher risk compared to the C allele (*p* = 0.004).

Conversely, the *IL5* rs2069812 variant showed no significant associations with asthma control or bronchodilator reversibility (Table 4). Moreover, the *IL4* rs2243250, *IL4* rs2243248, and *IL13* rs20541 genotypes did not exhibit any significant associations with the evaluated asthma phenotypes (S4–S6 Tables).

**Table 4:** Associations of IL5 rs2069812 genotype with asthma phenotype in asthma patients.

*IL5* rs2069812 genotype								
	Genotypes	N	Allergen sensitization (n, %)		PR (95% CI)	*p*-value		
			sIgE ≥ 0.35 kUA/l	sIgE < 0.35 kUA/l				
	CC	60	23 (38.9)	37 (56.0)		0.128	Ref.	
	CT	49	26 (44.1)	23 (34.8)			0.124	Ref.
	TT	16	10 (16.9)	6 (9.1)			0.083	0.509
	T allele	81	46 (38.9)	35 (26.5)	1.47 (1.02-2.11)	0.035*		
	C allele	169	72 (61.0)	97 (73.5)				
**Dominant pattern**	CT + TT	65	36 (61.0)	29 (43.9)	1.38 (0.99-1.95)	0.056		
	CC	60	23 (38.9)	37 (56.1)				
**Recessive pattern**	TT	16	10 (16.9)	6 (9.1)	1.86 (0.72-4.82)	0.189		
	CT + CC	109	49 (83.1)	60 (90.9)				
	**Genotypes**	**N**	**Blood eosinophilia (n, %)**		**PR (95% CI)**	***p*-value**		
			**EOS ≥ 150 Cells/uL**	**EOS < 150 Cells/uL**				
	CC	60	27 (33.3)	33 (75.0)		<0.0001**	Ref.	
	CT	49	41 (50.6)	8 (18.2)	3.09 (1.61-5.92)		<0.0001**	Ref.
	TT	16	13 (16.1)	3 (6.8)	3.9 (1.21-12.59)		0.009*	0.822
	T allele	81	67 (41.3)	14 (15.9)	2.59 (1.55-4.35)	<0.0001**		
	C allele	169	95 (58.6)	74 (84.1)				
**Dominant pattern**	CT + TT	65	54 (66.7)	11 (25.0)	2.67 (1.56-4.55)	<0.0001**		
	CC	60	27 (33.3)	33 (75.0)				
**Recessive pattern**	TT	16	13 (16.0)	3 (6.8)	2.35 (0.71-7.82)	0.14		
	CT + CC	109	68 (83.9)	41 (93.2)				
	**Genotypes**	**N**	**Asthma airflow limitation severity (n, %)**		**PR (95% CI)**	***p*-value**		
			**Pre-BD FEV**_**1**_ **< 70%**	**Pre-BD FEV**_**1**_ **≥ 70%**				
	CC	44	15 (35.7)	29 (63.0)		0.023*	Ref.	
	CT	32	21 (50.0)	11 (23.9)	2.12 (1.19-3.76)		0.006*	Ref.
	TT	12	6 (14.2)	6 (13.0)			0.313	0.343
	T allele	56	33 (39.3)	23 (25.0)	1.57 (1.01-2.45)	0.042*		
	C allele	120	51 (60.7)	69 (75.0)				
**Dominant pattern**	CT + TT	44	27 (64.3)	17 (36.9)	1.74 (1.12-2.69)	0.010*		
	CC	44	15 (35.7)	29 (63.0)				
**Recessive pattern**	TT	12	6 (14.3)	6 (13.0)	1.09 (0.38-3.13)	0.865		
	CT + CC	76	36 (85.7)	40 (86.9)				
	**Genotypes**	**N**	**Asthma controlled (n, %)**		**PR (95% CI)**	***p*-value**		
			**ACT score≤19**	**ACT score>19**				
	CC	53	11 (57.8)	42 (47.1)		0.732	Ref.	
	CT	40	6 (31.5)	34 (38.2)			0.477	Ref.
	TT	15	2 (10.5)	13 (14.6)			0.518	0.875
	T allele	70	10 (26.3)	60 (33.7)	0.78 (0.44-1.38)	0.376		
	C allele	146	28 (73.6)	118 (66.2)				
**Dominant pattern**	CT + TT	55	8 (42.1)	47 (52.8)	0.79 (0.45-1.39)	0.396		
	CC	53	11 (57.9)	42 (47.2)				
**Recessive pattern**	TT	15	2 (10.5)	13 (14.6)	0.72 (0.17-2.93)	0.641		
	CT + CC	93	17 (89.4)	76 (85.4)				
	**Genotypes**	**N**	**Bronchodilator reversibility (n, %)**		**PR (95% CI)**	***p*-value**		
			**FEV**_**1**_ **increase of <12% and <200 mL from baseline**	**FEV**_**1**_ **increase of ≥12% and ≥200 mL from baseline**				
	CC	32	29 (35.80%)	3 (50.00%)		0.724	Ref.	
	CT	44	41 (50.61%)	3 (50.00%)			0.683	Ref.
	TT	11	11 (13.59%)	0 (0.00%)			0.292	
	T allele	66	63 (38.88%)	3 (25.00%)	1.04 (0.96-1.12)			
	C allele	108	99 (61.12%)	9 (75.00%)				
**Dominant pattern**	CT + TT	55	52 (64.19%)	3 (50.00%)	1.04 (0.92-1.18)	0.486		
	CC	32	29 (35.81%)	3 (50.00%)				
**Recessive pattern**	TT	11	11 (13.58%)	0 (0.00%)	1.08 (1.02-1.16)	0.334		
	CT + CC	76	70 (86.42%)	6 (100.00%)				
	**Genotypes**	**N**	**Fixed airflow obstruction (n, %)**		**PR (95% CI)**	***p*-value**		
			**Post-BD FEV**_**1**_ **< 70%**	**Post-BD FEV1 ≥ 70%**				
	CC	60	25 (37.3)	35 (60.3)		0.023*	Ref.	
	CT	49	30 (44.7)	19 (32.7)	1.55 (1.00-2.39)		0.042*	Ref.
	TT	16	12 (17.9)	4 (6.9)	3.16 (1.12-8.93)		0.017*	0.317
	T allele	81	54 (40.3)	27 (20.7)	1.73 (1.17-2.55)	0.004*		
	C allele	169	80 (59.7)	89 (79.3)				
**Dominant pattern**	CT + TT	65	42 (62.7)	23 (39.6)	1.58 (1.09-2.28)	0.010*		
	CC	60	25 (37.3)	35 (60.3)				
**Recessive pattern**	TT	16	12 (17.9)	4 (6.9)	2.59 (0.89-7.61)	0.066		
	CT + CC	109	55 (82.1)	54 (93.1)				

*Nominal significance (*p* < 0.05), **Significance after Bonferroni correction (*p* < 1.25x10^-4^). N: Number of patients, PR: Prevalence ratio, sIgE: Specific-IgE, EOS: Eosinophils, ACT: Asthma control test, FEV1: Forced expiratory volume in one second, Pre-BD FEV_1_: pre-bronchodilator FEV_1_, Post-BD FEV_1_: post-bronchodilator FEV_1._

## Discussion

Both genetic and environmental factors play crucial roles in the pathogenesis of asthma. Allergic and eosinophilic inflammation are hallmark characteristics of type 2 predominant asthma subtypes [15,16]. Key cytokines, particularly interleukin (IL)-4, IL-13, and IL-5 are essential regulators of the immune responses that drive this pathophysiology. Specifically, IL-4 and IL-13 drive IgE-mediated mast cell degranulation, whereas IL-5 is central to eosinophil maturation and activation [17]. Fundamentally, asthma susceptibility is heavily influenced by genetics, with cohort studies estimating that up to 70% of the disease risk is inherited [18]. Despite numerous studies identifying asthma-associated polymorphisms, their precise functional impacts on distinct clinical and immunological phenotypes remain incompletely understood. In the present study, we initially investigated seven inflammatory cytokine gene polymorphisms: *IL4* (rs2243248 and rs2243250), *IL13* (rs20541 and rs1800925), and *IL5* rs2069812, and *IL33* (rs1342326 and rs3939286). Although *IL33* variants were targeted given the cytokine’s role as an upstream alarmin, they are excluded from subsequent analyses due to significant deviation from HWE. However, our findings reveal strong associations between the remaining five SNPs and specific asthma phenotypes, underscoring their critical involvement in immunological dysregulation, persistent eosinophilic inflammation, and overall disease severity.

The promoter SNP *IL4* rs2243248, located on chromosome 5q31-33, has been previously associated with asthma risk in the Asian population [19] and with mild asthma in Japanese individuals [7]. Functionally, the IL-4 cytokine regulates type 2 inflammation, in part, by inhibiting tumor necrosis factor-alpha (TNF-α) production via STAT6 signaling [20]. Interestingly, in our cohort, this SNP was significantly associated with TNF-α levels, with the TT genotype demonstrating the highest concentrations of this pro-inflammatory cytokine. TNF-α exerts multiple detrimental effects in the airways, including inducing the expression of other cytokines, chemokines, and adhesion molecules, all of which contribute to airway inflammation and hyperresponsiveness in asthma [21,22]. In contrast, the untranslated region (UTR) SNP *IL4* rs2243250, which has been implicated in asthma susceptibility in previous meta-analyses [23], did not exhibit any significant association with the clinical or immunological profiles in our investigation. This discrepancy suggests a potential ethnic-specific effect within the Thai population.

Regarding the *IL13* gene, the encoded IL-13 cytokine serves as a key mediator of type 2 inflammation by promoting dendritic cell activation, Th2 differentiation, and eosinophil recruitment [24]. The promoter polymorphism *IL13* rs1800925 has been well-documented as an asthma risk factor in Caucasian populations [8], and our findings robustly support its clinical relevance. Specifically, we observed that the TT genotype was significantly associated with both a higher percentage and a higher total count of Th2 cells, which aligns with the established role of IL-13 in amplifying type 2 immune responses [25]. Additionally, the T allele strongly correlated with an elevated risk of blood eosinophilia, thereby reinforcing its genetic contribution to eosinophil-mediated airway inflammation. Furthermore, we found that the CT genotype increased the risk of moderate asthma compared with the CC genotype, indicating that *IL13* polymorphisms may directly influence disease severity by driving eosinophilic inflammation [26]. Conversely, while the UTR SNP *IL13* rs20541 has been linked to asthma risk and airway hyperresponsiveness in Japanese adults [10], our study did not detect significant associations for this variant among Thai patients with asthma, further highlighting the potential for genetic heterogeneity across different populations.

The promoter polymorphism *IL5* rs2069812 crucially regulates gene expression levels, and its encoded cytokine, IL-5, plays a definitive role in eosinophil differentiation and the pathogenesis of eosinophilic asthma [27]. Previously, the TT genotype of *IL5* rs2069812 was identified as a risk factor for mild asthma and elevated eosinophil count in Iraqi patients [11]. Our findings strongly corroborate this relationship; we demonstrate that the TT genotype, the T allele, and the dominant inheritance model (CT + TT) were all significantly associated with increased blood eosinophil counts and a higher risk of clinical blood eosinophilia. Notably, this genotype-phenotype association remained robustly significant even after applying the stringent Bonferroni-adjusted threshold, underscoring the strength of this genetic link.

Interestingly, our findings regarding the severity of asthma airflow limitation diverge from previous reports that linked this variant primarily to mild asthma. In our cohort of Thai patients with asthma, the T allele, the CT genotype, and the dominant pattern were significantly associated with an increased risk of moderate-to-severe asthma. Crucially, to the best of our knowledge, the direct associations between *IL5* polymorphisms and the development of fixed airflow obstruction FAO or specific aeroallergen sensitization have not been previously reported. While earlier research linked *IL5* polymorphisms to baseline FEV_1_, and total IgE levels [28], and established that elevated IL-5 protein levels accelerate FEV1 decline, our study uniquely evaluated FAO using objective post-bronchodilator FEV1% predicted values. We discovered that the TT genotype, the CT genotype, the T allele, and the dominant pattern were all significant risk factors for FAO. This strongly implies that IL-5-driven eosinophilic inflammation may actively contribute to airway remodeling and persistent lung function decline. Furthermore, whereas previous genetic studies have primarily reported associations between *IL5* variants and total serum IgE levels [29], our comprehensive assessment revealed that the T allele also serves as a distinct risk factor for allergen sensitization through specific IgE (sIgE) production.

Despite the strength of these findings, several limitations warrant consideration. First, the cross-sectional design enables the identification of associations but precludes causal inference. Consequently, it remains unclear whether the investigated polymorphisms directly drive the observed phenotypes or if the development of FAO reflects cumulative long-term pathophysiological processes modulated by genetic background. Second, because the study population comprised exclusively Thai individuals, the generalizability of these results to other ethnicities may be limited. This is underscored by the lack of significant association for the *IL4* rs2243250 and *IL13* rs20541 variants in our cohort, despite their previously reported clinical relevance in other populations. Third, the study was designed to explore disease heterogeneity within an established cohort of patients with asthma; therefore, it did not include non-asthmatic healthy controls. Consequently, our findings cannot determine whether these variants confer initial susceptibility to developing asthma. Nevertheless, the data strongly indicate that specific genotypes, particularly within the *IL5* and *IL13* genes, predispose patients with established asthma to persistent eosinophilia and FAO, which are critical indicators of poor disease control and airway remodeling.

## Conclusion

Our study provides robust evidence that specific inflammatory cytokine gene polymorphisms serve as key determinants of clinically relevant asthma phenotypes. These insights significantly advance our understanding of the genetic architecture underlying asthma heterogeneity. Importantly, these findings highlight the potential clinical utility of utilizing the *IL5* rs2069812 and *IL13* rs1800925 variants as genetic biomarkers for disease risk stratification and the targeted application of precision biological therapies. Future longitudinal studies and mechanistic investigations across diverse populations are warranted to validate these findings and elucidate the precise molecular pathways driving these genotype-phenotype associations.

## Supporting information

S1 Table Hardy-Weinberg equilibrium (HWE) analysis of IL33 polymorphisms. The table presents the observed and expected genotype frequencies for IL33 rs1342326 and rs3939286 in the study population.
(DOCX)

S2 Table Comparison of clinical and immunological profiles according to interleukin genotypes IL4 rs2243248, IL13 rs1800925, and IL5 rs2069812 in asthma patients. The Kruskal-Wallis Test applied to the statistical analysis indicated significant differences among genotypes for various interleukin polymorphisms.
(DOCX)

S3 Table Comparison of clinical and immunological profiles according to interleukin genotypes IL4 rs2243250 and IL13 rs20541 in asthma patients. There were no statistically significant differences across several factors in the IL4 rs2243250 and IL13 rs20541 polymorphism genotypes in asthma patients.
(DOCX)

S4 Table Associations of IL4 rs2243250 genotype with asthma phenotype in asthma patients. This analysis revealed that no significant differences were observed in each interleukin polymorphism compared to the clinical outcomes in asthma patients using the Chi-square test.
(DOCX)

S5 Table Associations of IL4 rs2243248 genotype with asthma phenotype in asthma patients. The Chi-square test analysis of the IL4 rs2243248 genotype showed no significant differences in the polymorphism when compared to clinical outcomes in asthma patients.
(DOCX)

S6 Table Associations of IL13 rs20541 genotype with asthma phenotype in asthma patients. The Chi-square test analysis of the IL-13 rs20541 genotype showed no significant differences in the polymorphism compared to clinical outcomes in asthma patients.
(DOCX)

S1 File Minimal underlying dataset. Raw data of clinical, immunological, and spirometric parameters in asthma patients.
(XLSX)
