# Supplementary material for: IL5 rs2069812 and IL13 rs1800925 Genetic variants as key determinants of clinically relevant asthma phenotypes
Source: PLoS One. 2026 Jul 24;21(7):e0354597. doi: 10.1371/journal.pone.0354597 (PMC13399323; doi:10.1371/journal.pone.0354597)
Supplement: S1 Table — The table presents the observed and expected genotype frequencies for IL33 rs1342326 and rs3939286 in the study population. (DOCX) [file pone.0354597.s001.docx]

| **Gene positions** | **Genotype** | **Observed asthma patients** | **Expected asthma patients** | ***p*-value** |
| --- | --- | --- | --- | --- |
| *IL33* (rs1342326 A/C) | AA | 118 | 114.24 | 0.724 |
|  | AC | 3 | 10.52 | **0.02*** |
|  | CC | 4 | 0.24 | **<0.001**** |
| *IL33* (rs3939286 C/T) | CC | 97 | 80 | 0.057 |
|  | TC | 6 | 40 | **<0.001**** |
|  | TT | 22 | 5 | **<0.001**** |

**S1 Table.** Hardy-Weinberg equilibrium (HWE) analysis of *IL33* polymorphisms. The table presents the observed and expected genotype frequencies for *IL33* rs1342326 and rs3939286 in the study population. Both variants showed a highly significant deviation from HWE (*p* < 0.001), leading to their exclusion from subsequent genotype-phenotype association analyses. *Significant difference (*p* < 0.05), **Highly significant difference (*p* < 0.001).
