# Supplementary material for: IL5 rs2069812 and IL13 rs1800925 Genetic variants as key determinants of clinically relevant asthma phenotypes
Source: PLoS One. 2026 Jul 24;21(7):e0354597. doi: 10.1371/journal.pone.0354597 (PMC13399323; doi:10.1371/journal.pone.0354597)
Supplement: S2 Table — The Kruskal-Wallis Test applied to the statistical analysis indicated significant differences among genotypes for various interleukin polymorphisms. (DOCX) [file pone.0354597.s002.docx]

| **Parameters** | ***IL4* rs2243248 genotypes (mean ±SD; median, min-max)** | | | ***p*-value** | ***IL13* rs1800925 genotypes (mean ±SD; median, min-max)** | | | ***p*-value** | ***IL5* rs2069812 genotypes (mean ±SD; median, min-max)** | | | ***p*-value** |
| --- | --- | --- | --- | --- | --- | --- | --- | --- | --- | --- | --- | --- |
|  | **CC** | **CT** | **TT** |  | **CC** | **CT** | **TT** |  | **CC** | **CT** | **TT** |  |
|  | **N = 14 (11.20%)** | **N = 61 (48.80%)** | **N = 50 (40.00%)** |  | **N = 80 (64.00%)** | **N = 38 (30.40%)** | **N = 7 (5.60%)** |  | **N = 60 (48.00%)** | **N = 49 (39.20%)** | **N = 16 (12.80%)** |  |
| Specific-IgE (kUA/l) | 0.33 (0.03-50.50) | 0.44 (0.02-26.20) | 0.25 (0.03-101) | 0.521 | 0.25 (0.02-101) | 0.27 (0.02-50.5) | 0.55 (0.08-28.3) | 0.545 | 0.22 (0.02-101) | 0.44 (0.02-26.40) | 0.6 (0.03-50.50) | 0.112 |
| Cytokine levels (pg/ml) |  |  |  |  |  |  |  |  |  |  |  |  |
| Interleukin-4 | 0.05 (0-5.82) | 0 (0-93.12) | 0.02 (0-78.07) | 0.613 | 0 (0-93.12) | 0 (0-78.07) | 0 (0-36.34) | 0.826 | 0 (0-93.12) | 0 (0-78.07) | 0 (0-36.34) | 0.615 |
| Interleukin-5 | 0.10 (0-14.06) | 0 (0-17.16) | 1.03 (0-16.43) | 0.392 | 0.12 (0-17.16) | 0.54 (0-16.43) | 1.73 (0-4.4) | 0.633 | 0.12 (0-17.16) | 0.27 (0-6.99) | 1.44 (0-16.43) | 0.374 |
| Interleukin-6 | 1.41 (0-28) | 0 (0-31.65) | 1.6 (0-26.86) | 0.169 | 0.29 (0-26.86) | 0 (0-31.65) | 3.88 (0-28) | 0.161 | 0.47 (0-26.86) | 0 (0-31.65) | 3.67 (0-28) | 0.299 |
| Interleukin-10 | 0.01 (0-2.54) | 0.05 (0-16.46) | 0.03 (0-5.75) | 0.607 | 0.05 (0-16.46) | 0.01 (0-5.77) | 0.09 (0-1.01) | 0.511 | 0.05 (0-16.46) | 0 (0-3.03) | 0.06 (0-1.28) | 0.143 |
| Interleukin-13 | 0 (0-105.72) | 0 (0-153.85) | 0 (0-164.84) | 0.506 | 0 (0-164.84) | 0 (0-59.5) | 0 (0-0) | 0.753 | 0 (0-164.84) | 0 (0-55.55) | 0 (0-59.5) | 0.393 |
| TNF-α | 0 (0-35.83) | 0 (0-90.04) | 0.02 (0-128.77) | **0.038*** | 0 (0-90.04) | 0 (0-128.77) | 0 (0-10.79) | 0.366 | 0 (0-90.04) | 0 (0-128.77) | 0 (0-23.24) | 0.327 |
| Cell count (Cells/uL) |  |  |  |  |  |  |  |  |  |  |  |  |
| Blood eosinophil count | 195.5 (29-1077) | 288 (10-1635) | 191 (55-1116) | 0.966 | 177.5 (10-1116) | 275.5 (37-1561) | 218 (89-1635) | 0.092 | 135 (10-1077) | 272 (38-1561) | 316 (67-1635) | **<0.001**** |
| Th cells | 648 (272-1523) | 754 (227-2752) | 718 (172-1894) | 0.858 | 718 (172-1894) | 790.5 (272-2752) | 1207 (303-1933) | 0.285 | 691.5 (172-1501) | 797 (254-2752) | 771 (272-1933) | 0.112 |
| ILC2 cells | 123.5 (16-240) | 110 (16-476) | 104 (2-419) | 0.953 | 101.5 (2-419) | 115.5 (16-340) | 167 (34-476) | 0.289 | 98 (2-419) | 109 (16-394) | 137.5 (16-476) | 0.766 |
| Th2 cells | 250.5 (2-692) | 151 (0-1567) | 77.5 (0-1311) | 0.352 | 93 (0-1131) | 136 (0-1311) | 625 (234-1567) | **0.011*** | 153.5 (0-1118) | 70 (0-1176) | 263 (0-1567) | 0.178 |
| % Th of Lymphocytes | 36.9 (23-55.2) | 36 (18.3-52.6) | 38.4 (16.6-64.7) | 0.142 | 37.4 (16.6-64.7) | 36.7 (18.6-50.1) | 32.2 (31.3-49.9) | 0.684 | 34.7 (16.6-53.3) | 37.7 (18.3-64.7) | 37.3 (28.2-49.9) | 0.389 |
| % ILC2 of Th cells | 9.6 (2-31.1) | 10.5 (1.7-64.6) | 10 (1.2-32.8) | 0.669 | 9.8 (1.2-33.6) | 10.2 (2.1-64.6) | 12.6 (6.4-23.6) | 0.592 | 10 (1.2-32.8) | 10.1 (1.7-64.6) | 10.3 (3.6-33.7) | 0.957 |
| % Th2 of Th cells | 19.4 (0.1-99) | 22.6 (0-99.7) | 8.5 (0-95) | 0.233 | 9 (0-99) | 14.4 (0-99.7) | 65.8 (31-96.1) | **0.025*** | 23.4 (0-99) | 4.6 (0-99.5) | 42.8 (0-99.7) | 0.111 |
| Spirometry |  |  |  |  |  |  |  |  |  |  |  |  |
| Pre-BD FEV1 (%pred) | 69.66±16.35 | 68.1±23.77 | 63.24±24.18 | 0.531 | 68.43±20.65 | 58.19±24.02 | 83.6±33.32 | 0.079 | 72.15±17.68 | 56.91±25.04 | 70.58±27.81 | **0.033*** |
| Post-BD FEV1 (%pred) | 73.85±15.53 | 65.91±26.12 | 67.8±22.26 | 0.628 | 68.96±22.91 | 65±21.50 | 65.42±40.97 | 0.261 | 72.18±21.81 | 65.35±20.40 | 57±34.39 | **0.012*** |
| Pre-BD FVC (%pred) | 75.5±25.55 | 79.15±23.57 | 81±15.99 | 0.891 | 79.96±18.06 | 75.52±25.34 | 92.6±25.39 | 0.518 | 82.41±19.34 | 74.96±18.72 | 80.36±30.86 | 0.231 |
| Post-BD FVC (%pred) | 81.5±13.62 | 82.46±19.21 | 80.75±19.38 | 0.716 | 81.68±17.02 | 82.44±14.44 | 76.8±44.46 | 0.984 | 84.02±18.45 | 78.96±13.18 | 78.72±29.52 | 0.252 |
| FEV_1_/FVC ratio | 0.73±0.11 | 0.69±0.13 | 0.71±0.12 | 0.632 | 0.72±0.11 | 0.67±0.15 | 0.72±0.07 | 0.587 | 0.72±0.13 | 0.67±0.12 | 0.73±0.13 | 0.124 |
| %ΔFEV1 (%) | 5.93 (-2.77-20.16) | 5.18 (-7.69-36) | 8.73 (-3.65-79.48) | 0.303 | 5.23 (-3.65-36) | 7.06 (-7.69-79.48) | 8.40 (2.38-11.71) | 0.543 | 5.23 (-3.65-36) | 6.61 (-7.69-31.73) | 7.62 (-2.77-79.48) | 0.786 |

**S2 Table.** Comparison of clinical and immunological profiles according to interleukin genotypes *IL4* rs2243248, *IL13* rs1800925, and *IL5* rs2069812 in asthma patients. The Kruskal-Wallis Test applied to the statistical analysis indicated significant differences among genotypes for various interleukin polymorphisms. For the *IL4* rs2243248 polymorphism, significant differences were observed in TNF-α levels (*p* = 0.038), with the TT genotype exhibiting the highest TNF-α levels compared to the CC and CT genotypes. In the *IL13* rs1800925 polymorphism, significant differences were found in Th2 cell counts (*p* = 0.011) and the percentage of Th2 cells among Th cells (*p* = 0.025), with the TT genotype demonstrating the highest counts compared to the CC and CT genotypes. For the *IL5* rs2069812, significant differences were observed in blood eosinophil counts (*p* < 0.001), pre-bronchodilator FEV1 %predicted (*p* = 0.033), and post-bronchodilator FEV1 %predicted (*p* = 0.012)

*Significant difference (*p* < 0.05), **Highly significant difference (*p* < 0.001). N: Number of patients, Th: T helper cell, ILC2: Type 2 innate lymphoid cells, Th2: T helper two cells, Pre-BD: pre-bronchodilator, Post-BD: post-bronchodilator, FEV_1_: Forced expiratory volume in one second, FVC: Forced vital capacity.
