# Supplementary material for: IL5 rs2069812 and IL13 rs1800925 Genetic variants as key determinants of clinically relevant asthma phenotypes
Source: PLoS One. 2026 Jul 24;21(7):e0354597. doi: 10.1371/journal.pone.0354597 (PMC13399323; doi:10.1371/journal.pone.0354597)
Supplement: S3 Table — There were no statistically significant differences across several factors in the IL4 rs2243250 and IL13 rs20541 polymorphism genotypes in asthma patients. (DOCX) [file pone.0354597.s003.docx]

| **Parameters** | ***IL4* rs2243250 genotypes (mean ±SD; median, min-max)** | | | ***p*-value** | ***IL13* rs20541 genotypes (mean ±SD; median, min-max)** | | | ***p*-value** |
| --- | --- | --- | --- | --- | --- | --- | --- | --- |
|  | **CC** | **CT** | **TT** |  | **AA** | **AG** | **GG** |  |
|  | **N = 15 (12.00%)** | **N = 59 (47.20%)** | **N = 51 (40.80%)** |  | **N = 20 (16.00%)** | **N = 63 (50.40%)** | **N = 42 (33.60%)** |  |
| Specific-IgE (kUA/l) | 0.42 (0.03-50.50) | 0.49 (0.02-26.20) | 0.22 (0.02-101) | 0.395 | 0.42 (0.03-101) | 0.33 (0.02-50.5) | 0.21 (0.02-20.4) | 0.762 |
| Cytokine levels (pg/ml) | | | | |  | | | |
| Interleukin-4 | 0 (0-5.82) | 0 (0-93.12) | 0 (0-78.07) | 0.841 | 0 (0-36.34) | 0 (0-78.07) | 0.3 (0-93.12) | 0.125 |
| Interleukin-5 | 0 (0-14.06) | 0 (0-17.16) | 0.92 (0-16.43) | 0.376 | 1.38 (0-6.99) | 0 (0-16.43) | 0.04 (0-17.16) | 0.141 |
| Interleukin-6 | 2.83 (0-31.65) | 0 (0-22.28) | 1.67 (0-26.86) | 0.059 | 0 (0-28) | 0 (0-31.65) | 1.29 (0-22.28) | 0.569 |
| Interleukin-10 | 0 (0-2.54) | 0.09 (0-16.46) | 0.03 (0-5.75) | 0.435 | 0.03 (0-1.72) | 0.03 (0-16.46) | 0.03 (0-4.23) | 0.957 |
| Interleukin-13 | 0 (0-105.72) | 0 (0-153.85) | 0 (0-164.84) | 0.544 | 0 (0-42.48) | 0 (0-164.84) | 0 (0-153.85) | 0.953 |
| TNF-α | 0 (0-35.83) | 0 (0-90.04) | 0 (0-128.77) | 0.111 | 0 (0-32.74) | 0 (0-128.77) | 0 (0-90.04) | 0.346 |
| Cell count (Cells/uL) | | | | |  | | | |
| Blood eosinophil count | 224 (29-1077) | 204 (10-1635) | 194 (55-1116) | 0.993 | 298.5 (89-1635) | 204 (37-1561) | 182 (10-1077) | 0.068 |
| Th cells | 698 (272-1523) | 754 (227-2752) | 716 (172-1894) | 0.931 | 909 (254-1933) | 710 (272-2752) | 700.5 (172-1413) | 0.094 |
| ILC2 cells | 138 (16-240) | 112 (16-476) | 99 (2-419) | 0.825 | 151 (34-476) | 107 (16-340) | 87 (2-419) | 0.154 |
| Th2 cells | 305 (2-692) | 151 (0-1567) | 70 (0-1311) | 0.291 | 216.5 (0-1567) | 148 (0-1311) | 95.5 (0-1131) | 0.49 |
| % Th of Lymphocytes | 36.5 (23-55.2) | 35.4 (18.3-52.6) | 38.6 (16.6-64.7) | 0.087 | 36.9 (23.9-49.9) | 36.9 (18.6-50.6) | 36.8 (16.6-64.7) | 0.996 |
| % ILC2 of Th cells | 10 (2-31.1) | 10.4 (1.7-64.6) | 10 (1.2-32.8) | 0.838 | 12 (2.1-33.7) | 9.6 (1.5-64.6) | 9.7 (1.2-33.6) | 0.313 |
| % Th2 of Th cells | 23.7 (0.1-99) | 22.6 (0-99.7) | 7.9 (0-95) | 0.194 | 23.7 (0-99.7) | 14.7 (0-99.5) | 11.9 (0-99) | 0.742 |
| Spirometry | | | | |  | | | |
| Pre-BD FEV1 (%pred) | 70.69±16.09 | 68.36±24.27 | 62.56±23.71 | 0.314 | 67.78±25.68 | 65.81±24.25 | 66.28±20.73 | 0.791 |
| Post-BD FEV1 (%pred) | 74.86±15.46 | 66.05±26.62 | 67.15±21.75 | 0.477 | 68.2±31.51 | 67.238±20.21 | 67.74±24.63 | 0.784 |
| Pre-BD FVC (%pred) | 77.31±25.32 | 79.44±23.78 | 80.14±15.88 | 0.851 | 82.07±19.85 | 81.31±22.61 | 75.81±19.08 | 0.276 |
| Post-BD FVC (%pred) | 82.76±13.83 | 82.89±19.09 | 79.86±19.49 | 0.533 | 78.35±26.86 | 85.73±14.05 | 77.78±18.54 | 0.194 |
| FEV_1_/FVC ratio | 0.74±0.11 | 0.69±0.13 | 0.71±0.11 | 0.528 | 0.68±0.09 | 0.70±0.14 | 0.72±0.11 | 0.565 |
| %ΔFEV1 (%) | 6.61 (-2.77-20.16) | 5.16 (-3.57-36) | 8.73 (-7.69-79.48) | 0.398 | 8.53 (-3-31.73) | 5.69 (-7.69-79.48) | 5.64 (-3.65-19.27) | 0.371 |

**S3 Table.** Comparison of clinical and immunological profiles according to interleukin genotypes *IL4* rs2243250 and *IL13* rs20541 in asthma patients. There were no statistically significant differences across several factors in the *IL4* rs2243250 and *IL13* rs20541 polymorphism genotypes in asthma patients.

N: Number of patients, Th: T helper cell, ILC2: Type 2 innate lymphoid cells, Th2: T helper two cells, Pre-BD: pre-bronchodilator, Post-BD: post-bronchodilator, FEV_1_: Forced expiratory volume in one second, FVC: Forced vital capacity.
