# Supplementary material for: IL5 rs2069812 and IL13 rs1800925 Genetic variants as key determinants of clinically relevant asthma phenotypes
Source: PLoS One. 2026 Jul 24;21(7):e0354597. doi: 10.1371/journal.pone.0354597 (PMC13399323; doi:10.1371/journal.pone.0354597)
Supplement: S4 Table — This analysis revealed that no significant differences were observed in each interleukin polymorphism compared to the clinical outcomes in asthma patients using the Chi-square test. (DOCX) [file pone.0354597.s004.docx]

| ***IL4* rs2243250 genotype** | | | | | | | | |
| --- | --- | --- | --- | --- | --- | --- | --- | --- |
|  | **Genotypes** | **N** | **Allergen sensitization (n, %)** | | **PR (95% CI)** | ***p*-value** |  |  |
|  |  |  | **sIgE≥0.35 kUA/l** | **sIgE<0.35 kUA/l** |  |  |  |  |
|  | CC | 15 | 8 (13.5) | 7 (10.6) |  | 0.346 | Ref. |  |
|  | CT | 59 | 31 (52.5) | 28 (42.4) |  |  | 0.956 | Ref. |
|  | TT | 51 | 20 (33.9) | 31 (46.9) |  |  | 0.331 | 0.162 |
|  | T allele | 161 | 71 (60.1) | 90 (68.1) | 0.88 (0.73-1.06) | 0.186 |  |  |
|  | C allele | 89 | 47 (39.8) | 42 (31.8) |  |  |  |  |
| **Dominant pattern** | CT+TT | 110 | 51 (86.4) | 59 (89.3) | 0.97 (0.84-1.10) | 0.612 |  |  |
|  | CC | 15 | 8 (13.5) | 7 (10.6) |  |  |  |  |
| **Recessive pattern** | TT | 51 | 20 (33.8) | 31 (46.9) | 0.72 (0.46-1.12) | 0.138 |  |  |
|  | CT+CC | 74 | 39 (66.1) | 35 (53.0) |  |  |  |  |
|  | **Genotypes** | **N** | **Blood eosinophilia (n, %)** | | **PR (95% CI)** | ***p*-value** |  |  |
|  |  |  | **EOS≥150 Cells/uL** | **EOS<150 Cells/uL** |  |  |  |  |
|  | CC | 15 | 11 (13.5) | 4 (9.1) |  | 0.506 | Ref. |  |
|  | CT | 59 | 40 (49.3) | 19 (43.1) |  |  | 0.679 | Ref. |
|  | TT | 51 | 30 (37.0) | 21 (47.7) |  |  | 0.308 | 0.329 |
|  | T allele | 161 | 100 (61.7) | 61 (69.3) | 0.89 (0.74-1.07) | 0.231 |  |  |
|  | C allele | 89 | 62 (38.2) | 27 (30.6) |  |  |  |  |
| **Dominant pattern** | CT+TT | 110 | 70 (86.4) | 40 (90.9) | 0.95 (0.84-1.08) | 0.461 |  |  |
|  | CC | 15 | 11 (13.5) | 4 (9.0) |  |  |  |  |
| **Recessive pattern** | TT | 51 | 30 (37.0) | 21 (47.7) | 0.78 (0.51-1.18) | 0.245 |  |  |
|  | CT+CC | 74 | 51 (62.9) | 23 (52.2) |  |  |  |  |
|  | **Genotypes** | **N** | **Asthma severity (n, %)** | | **PR (95% CI)** | ***p*-value** |  |  |
|  |  |  | **Pre-BD FEV_1_<70%** | **Pre-BD FEV1≥70%** |  |  |  |  |
|  | CC | 13 | 4 (9.5) | 9 (15.5) |  | 0.241 | Ref. |  |
|  | CT | 38 | 17 (40.4) | 21 (45.6) |  |  | 0.377 | Ref. |
|  | TT | 37 | 21 (50.0) | 16 (38.7) |  |  | 0.106 | 0.297 |
|  | T allele | 112 | 59 (70.2) | 53 (57.6) | 1.22 (0.97-1.52) | 0.081 |  |  |
|  | C allele | 64 | 25 (29.7) | 39 (42.3) |  |  |  |  |
| **Dominant pattern** | CT+TT | 75 | 38 (90.4) | 37 (80.4) | 1.12 (0.95-1.34) | 0.184 |  |  |
|  | CC | 13 | 4 (9.5) | 9 (19.5) |  |  |  |  |
| **Recessive pattern** | TT | 37 | 21 (50.0) | 16 (34.7) | 1.44 (0.87-2.36) | 0.148 |  |  |
|  | CT+CC | 51 | 21 (50.0) | 30 (65.2) |  |  |  |  |
|  | **Genotypes** | **N** | **Asthma controlled (n, %)** | | **PR (95% CI)** | ***p*-value** |  |  |
|  |  |  | **ACT score≤19** | **ACT score>19** |  |  |  |  |
|  | CC | 15 | 1 (5.2) | 14 (15.7) |  | 0.510 | Ref. |  |
|  | CT | 51 | 9 (47.3) | 42 (47.1) |  |  | 0.297 | Ref. |
|  | TT | 42 | 9 (47.3) | 33 (37.0) |  |  | 0.196 | 0.646 |
|  | T allele | 135 | 27 (71.0) | 108 (60.6) | 1.17 (0.92-1.48) | 0.23 |  |  |
|  | C allele | 81 | 11 (28.9) | 70 (39.3) |  |  |  |  |
| **Dominant pattern** | CT+TT | 93 | 18 (94.7) | 75 (84.2) | 1.12 (0.97-1.29) | 0.231 |  |  |
|  | CC | 15 | 1 (5.2) | 14 (15.7) |  |  |  |  |
| **Recessive pattern** | TT | 42 | 9 (47.3) | 33 (37.0) | 1.27 (0.74-2.20) | 0.403 |  |  |
|  | CT+CC | 66 | 10 (52.6) | 56 (62.9) |  |  |  |  |
|  | **Genotypes** | **N** | **Bronchodilator reversibility (n, %)** | | **PR (95% CI)** | ***p*-value** |  |  |
|  |  |  | **FEV_1_ increase of <12% and <200 mL from baseline** | **FEV_1_ increase of ≥12% and ≥200 mL from baseline** |  |  |  |  |
|  | CC | 13 | 2 (10.0) | 11 (15.9) |  | 0.172 | Ref. |  |
|  | CT | 39 | 6 (30.0) | 33 (47.8) |  |  | 1 | Ref. |
|  | TT | 37 | 12 (60.0) | 25 (36.2) |  |  | 0.238 | 0.081 |
|  | T allele | 113 | 30 (75.0) | 83 (60.1) | 1.24 (0.99-1.56) | 0.085 |  |  |
|  | C allele | 65 | 10 (25.0) | 55 (39.8) |  |  |  |  |
| **Dominant pattern** | CT+TT | 76 | 18 (90.0) | 58 (84.0) | 1.07 (0.89-1.28) | 0.507 |  |  |
|  | CC | 13 | 2 (10.0) | 11 (15.9) |  |  |  |  |
| **Recessive pattern** | TT | 37 | 12 (60.0) | 25 (36.2) | 1.65 (1.02-2.66) | 0.057 |  |  |
|  | CT+CC | 52 | 8 (40.0) | 44 (63.7) |  |  |  |  |
|  | **Genotypes** | **N** | **Fixed airflow obstruction (n, %)** | | **PR (95% CI)** | ***p*-value** |  |  |
|  |  |  | **Post-BD FEV_1_<70%** | **Post-BD FEV1≥70%** |  |  |  |  |
|  | CC | 15 | 5 (7.4) | 10 (17.2) |  | 0.219 | Ref. |  |
|  | CT | 59 | 32 (47.7) | 27 (46.5) |  |  | 0.148 | Ref. |
|  | TT | 51 | 30 (44.7) | 21 (36.2) |  |  | 0.082 | 0.628 |
|  | T allele | 161 | 92 (68.6) | 69 (59.4) | 1.15 (0.95-1.39) | 0.131 |  |  |
|  | C allele | 89 | 42 (31.3) | 47 (40.5) |  |  |  |  |
| **Dominant pattern** | CT+TT | 110 | 62 (92.5) | 48 (82.7) | 1.12 (0.97-1.28) | 0.093 |  |  |
|  | CC | 15 | 5 (7.4) | 10 (17.2) |  |  |  |  |
| **Recessive pattern** | TT | 51 | 30 (44.7) | 21 (36.2) | 1.23 (0.80-1.91) | 0.331 |  |  |
|  | CT+CC | 74 | 37 (55.2) | 37 (63.7) |  |  |  |  |

**S4 Table.** Associations of *IL4* rs2243250 genotype with asthma phenotype in asthma patients. *IL4* rs2243250 genotype analyzed by the Chi-square test. This analysis revealed that no significant differences were observed in each interleukin polymorphism compared to the clinical outcomes in asthma patients.

*Nominal significance (*p* < 0.05), **Significance after Bonferroni correction (*p* < 1.25x10^-4^). N: Number of patients, PR: Prevalence ratio, sIgE: Specific-IgE, EOS: Eosinophils, ACT: Asthma control test, FEV1: Forced expiratory volume in one second, Pre-BD FEV1: pre-bronchodilator FEV1, Post-BD FEV1: post-bronchodilator FEV1.
