# Supplementary material for: IL5 rs2069812 and IL13 rs1800925 Genetic variants as key determinants of clinically relevant asthma phenotypes
Source: PLoS One. 2026 Jul 24;21(7):e0354597. doi: 10.1371/journal.pone.0354597 (PMC13399323; doi:10.1371/journal.pone.0354597)
Supplement: S5 Table — The Chi-square test analysis of the IL4 rs2243248 genotype showed no significant differences in the polymorphism when compared to clinical outcomes in asthma patients. (DOCX) [file pone.0354597.s005.docx]

| ***IL4* rs2243248 genotype** | | | | | | | | |
| --- | --- | --- | --- | --- | --- | --- | --- | --- |
|  | **Genotypes** | **N** | **Allergen sensitization (n, %)** | | **PR (95% CI)** | ***p*-value** |  |  |
|  |  |  | **sIgE≥0.35 kUA/l** | **sIgE<0.35 kUA/l** |  |  |  |  |
|  | CC | 14 | 7 (11.8) | 7 (10.6) |  | 0.624 | Ref. |  |
|  | CT | 61 | 31 (52.5) | 30 (45.4) |  |  | 0.955 | Ref. |
|  | TT | 50 | 21 (35.) | 29 (43.9) |  |  | 0.593 | 0.354 |
|  | T allele | 161 | 73 (61.8) | 88 (66.6) | 0.93 (0.77-1.12) | 0.428 |  |  |
|  | C allele | 89 | 45 (38.1) | 44 (33.3) |  |  |  |  |
| **Dominant pattern** | CT+TT | 111 | 52 (88.1) | 59 (89.3) | 0.98 (0.86-1.12) | 0.823 |  |  |
|  | CC | 14 | 7 (11.8) | 7 (10.6) |  |  |  |  |
| **Recessive pattern** | TT | 50 | 21 (35.5) | 29 (43.9) | 0.81 (0.52-1.25) | 0.342 |  |  |
|  | CT+CC | 75 | 38 (64.4) | 37 (56.0) |  |  |  |  |
|  | **Genotypes** | **N** | **Blood eosinophilia (n, %)** | | **PR (95% CI)** | ***p*-value** |  |  |
|  |  |  | **EOS≥150 Cells/uL** | **EOS<150 Cells/uL** |  |  |  |  |
|  | CC | 14 | 10 (12.3) | 4 (9.0) |  | 0.445 | Ref. |  |
|  | CT | 61 | 42 (51.8) | 19 (43.1) |  |  | 0.851 | Ref. |
|  | TT | 50 | 29 (35.8) | 21 (47.7) |  |  | 0.362 | 0.236 |
|  | T allele | 161 | 100 (61.7) | 61 (69.3) | 0.89 (0.74-1.07) | 0.231 |  |  |
|  | C allele | 89 | 62 (38.2) | 27 (30.6) |  |  |  |  |
| **Dominant pattern** | CT+TT | 111 | 71 (87.6) | 40 (90.9) | 0.96 (0.85-1.09) | 0.582 |  |  |
|  | CC | 14 | 10 (12.3) | 4 (9.0) |  |  |  |  |
| **Recessive pattern** | TT | 50 | 29 (35.8) | 21 (47.7) | 0.75 (0.49-1.15) | 0.194 |  |  |
|  | CT+CC | 75 | 52 (64.2) | 23 (52.2) |  |  |  |  |
|  | **Genotypes** | **N** | **Asthma airflow limitation severity (n, %)** | | **PR (95% CI)** | ***p*-value** |  |  |
|  |  |  | **Pre-BD FEV_1_<70%** | **Pre-BD FEV1≥70%** |  |  |  |  |
|  | CC | 12 | 4 (9.5) | 8 (17.3) |  | 0.449 | Ref. |  |
|  | CT | 39 | 18 (42.8) | 21 (45.6) |  |  | 0.432 | Ref. |
|  | TT | 37 | 20 (47.6) | 17 (36.9) |  |  | 0.212 | 0.491 |
|  | T allele | 113 | 58 (69.0) | 55 (59.7) | 1.15 (0.93-1.44) | 0.2 |  |  |
|  | C allele | 63 | 26 (30.9) | 37 (40.2) |  |  |  |  |
| **Dominant pattern** | CT+TT | 76 | 38 (90.4) | 38 (82.6) | 1.09 (0.92-1.29) | 0.282 |  |  |
|  | CC | 12 | 4 (9.5) | 8 (17.4) |  |  |  |  |
| **Recessive pattern** | TT | 37 | 20 (90.9) | 17 (36.9) | 1.29 (0.78-2.11) | 0.311 |  |  |
|  | CT+CC | 51 | 22 (9.1) | 29 (63.0) |  |  |  |  |
|  | **Genotypes** | **N** | **Asthma controlled (n, %)** | | **PR (95% CI)** | ***p*-value** |  |  |
|  |  |  | **ACT score≤19** | **ACT score>19** |  |  |  |  |
|  | CC | 14 | 1 (5.2) | 13 (14.6) |  | 0.565 | Ref. |  |
|  | CT | 53 | 9 (47.3) | 44 (49.4) |  |  | 0.358 | Ref. |
|  | TT | 41 | 9 (47.3) | 32 (35.9) |  |  | 0.214 | 0.543 |
|  | T allele | 135 | 27 (71.0) | 108 (60.6) | 1.17 (0.93-1.48) | 0.23 |  |  |
|  | C allele | 81 | 11 (28.9) | 70 (39.3) |  |  |  |  |
| **Dominant pattern** | CT+TT | 94 | 18 (94.7) | 76 (85.3) | 1.11 (0.96-1.27) | 0.271 |  |  |
|  | CC | 14 | 1 (5.2) | 13 (14.6) |  |  |  |  |
| **Recessive pattern** | TT | 41 | 9 (47.3) | 32 (35.9) | 1.32 (0.76-2.28) | 0.352 |  |  |
|  | CT+CC | 67 | 10 (52.6) | 57 (64.0) |  |  |  |  |
|  | **Genotypes** | **N** | **Bronchodilator reversibility (n, %)** | | **PR (95% CI)** | ***p*-value** |  |  |
|  |  |  | **FEV_1_ increase of <12% and <200 mL from baseline** | **FEV_1_ increase of ≥12% and ≥200 mL from baseline** |  |  |  |  |
|  | CC | 12 | 2 (10.0) | 10 (14.4) |  | 0.176 | Ref. |  |
|  | CT | 40 | 6 (30.0) | 34 (49.2) |  |  | 0.888 | Ref. |
|  | TT | 37 | 12 (60.0) | 25 (36.2) |  |  | 0.293 | 0.071 |
|  | T allele | 114 | 30 (75.0) | 84 (60.8) | 1.23 (0.95-1.54) | 0.101 |  |  |
|  | C allele | 64 | 10 (25.0) | 54 (39.1) |  |  |  |  |
| **Dominant pattern** | CT+TT | 77 | 18 (90.0) | 59 (85.5) | 1.05 (0.88-1.25) | 0.604 |  |  |
|  | CC | 12 | 2 (10.0) | 10 (14.4) |  |  |  |  |
| **Recessive pattern** | TT | 37 | 12 (60.0) | 25 (36.2) | 1.65 (1.02-2.66) | 0.057 |  |  |
|  | CT+CC | 52 | 8 (40.0) | 44 (63.7) |  |  |  |  |
|  | **Genotypes** | **N** | **Fixed airflow obstruction (n, %)** | | **PR (95% CI)** | ***p*-value** |  |  |
|  |  |  | **Post-BD FEV_1_<70%** | **Post-BD FEV1≥70%** |  |  |  |  |
|  | CC | 14 | 5 (7.4) | 9 (15.5) |  | 0.388 | Ref. |  |
|  | CT | 61 | 34 (50.7) | 27 (46.5) |  |  | 0.176 | Ref. |
|  | TT | 50 | 28 (41.8) | 22 (37.9) |  |  | 0.179 | 0.977 |
|  | T allele | 161 | 90 (67.1) | 71 (61.2) | 1.09 (0.91-1.32) | 0.326 |  |  |
|  | C allele | 89 | 44 (32.8) | 45 (38.7) |  |  |  |  |
| **Dominant pattern** | CT+TT | 111 | 62 (92.5) | 49 (84.4) | 1.09 (0.96-1.24) | 0.154 |  |  |
|  | CC | 14 | 5 (7.4) | 9 (15.5) |  |  |  |  |
| **Recessive pattern** | TT | 50 | 28 (41.7) | 22 (37.9) | 1.10 (0.71-1.70) | 0.66 |  |  |
|  | CT+CC | 75 | 39 (58.2) | 36 (62.0) |  |  |  |  |

**S5** **Table.** Associations of *IL4* rs2243248 genotype with asthma phenotype in asthma patients. The Chi-square test analysis of the *IL4* rs2243248 genotype showed no significant differences in the polymorphism when compared to clinical outcomes in asthma patients

*Nominal significance (*p* < 0.05), **Significance after Bonferroni correction (*p* < 1.25x10^-4^). N: Number of patients, PR: Prevalence ratio, sIgE: Specific-IgE, EOS: Eosinophils, ACT: Asthma control test, FEV1: Forced expiratory volume in one second, Pre-BD FEV1: pre-bronchodilator FEV1, Post-BD FEV1: post-bronchodilator FEV1._._
