# Supplementary material for: IL5 rs2069812 and IL13 rs1800925 Genetic variants as key determinants of clinically relevant asthma phenotypes
Source: PLoS One. 2026 Jul 24;21(7):e0354597. doi: 10.1371/journal.pone.0354597 (PMC13399323; doi:10.1371/journal.pone.0354597)
Supplement: S6 Table — The Chi-square test analysis of the IL-13 rs20541 genotype showed no significant differences in the polymorphism compared to clinical outcomes in asthma patients. (DOCX) [file pone.0354597.s006.docx]

| ***IL13* rs20541 genotype** | | | | | | | | | | |
| --- | --- | --- | --- | --- | --- | --- | --- | --- | --- | --- |
|  | **Genotypes** | **N** | **Allergen sensitization (n, %)** | | **PR (95% CI)** | ***p*-value** | |  | |  |
|  |  |  | **sIgE≥0.35 kUA/l** | **sIgE<0.35 kUA/l** |  |  |  |  |  |  |
|  | AA | 20 | 10 (16.9) | 10 (15.1) |  | 0.833 | | Ref. | |  |
|  | AG | 63 | 31 (52.5) | 32 (48.4) |  |  | | 0.951 | | Ref. |
|  | GG | 42 | 18 (30.5) | 24 (36.3) |  |  | | 0.597 | | 0.523 |
|  | G allele | 147 | 67 (56.7) | 80 (60.6) | 0.94 (0.76-1.15) | 0.539 | |  | |  |
|  | A allele | 103 | 51 (43.2) | 52 (39.3) |  |  | |  | |  |
| **Dominant pattern** | AG+AA | 83 | 41 (69.4) | 42 (63.6) | 1.09 (0.85-1.40) | 0.489 | |  | |  |
|  | GG | 42 | 18 (30.5) | 24 (36.3) |  |  | |  | |  |
| **Recessive pattern** | AA | 20 | 10 (16.9) | 10 (10.6) | 1.11 (0.50-2.49) | 0.784 | |  | |  |
|  | AG+GG | 105 | 49 (83.0) | 56 (89.3) |  |  | |  | |  |
|  | **Genotypes** | **N** | **Blood eosinophilia (n, %)** | | **PR (95% CI)** | ***p*-value** | |  | |  |
|  |  |  | **EOS≥150 Cells/uL** | **EOS<150 Cells/uL** |  |  |  |  |  |  |
|  | AA | 20 | 16 (19.7) | 4 (9.1) |  | 0.213 | | Ref. | |  |
|  | AG | 63 | 41 (50.6) | 22 (50.0) |  |  | | 0.21 | | Ref. |
|  | GG | 42 | 24 (29.6) | 18 (40.9) |  |  | | 0.078 | | 0.412 |
|  | G allele | 147 | 89 (54.9) | 58 (65.9) | 0.83 (0.68-1.02) | 0.092 | |  | |  |
|  | A allele | 103 | 73 (45.0) | 30 (34.1) |  |  | |  | |  |
| **Dominant pattern** | AG+AA | 83 | 57 (70.3) | 26 (59.1) | 1.19 (0.89-1.58) | 0.202 | |  | |  |
|  | GG | 42 | 24 (29.6) | 18 (40.9) |  |  | |  | |  |
| **Recessive pattern** | AA | 20 | 16 (19.7) | 4 (9.0) | 2.17 (0.77-6.10) | 0.12 | |  | |  |
|  | AG+GG | 105 | 65 (80.2) | 40 (90.9) |  |  | |  | |  |
|  | **Genotypes** | **N** | **Asthma airflow limitation severity (n, %)** | | **PR (95% CI)** | ***p*-value** | |  | |  |
|  |  |  | **Pre-BD FEV_1_<70%** | **Pre-BD FEV1≥70%** |  |  |  |  |  |  |
|  | AA | 14 | 9 (21.4) | 5 (10.8) |  | 0.405 | | Ref. | |  |
|  | AG | 43 | 20 (47.6) | 23 (50.0) |  |  | | 0.247 | | Ref. |
|  | GG | 31 | 13 (30.9) | 18 (39.1) |  |  | | 0.165 | | 0.696 |
|  | G allele | 105 | 46 (54.7) | 59 (64.1) | 0.85 (0.66-1.09) | 0.205 | |  | |  |
|  | A allele | 71 | 38 (45.2) | 33 (35.8) |  |  | |  | |  |
| **Dominant pattern** | AG+AA | 57 | 29 (69.0) | 28 (60.8) | 1.13 (0.83-1.54) | 0.422 | |  | |  |
|  | GG | 31 | 13 (30.9) | 18 (39.1) |  |  | |  | |  |
| **Recessive pattern** | AA | 14 | 9 (21.4) | 5 (10.8) | 1.97 (0.71-5.41) | 0.176 | |  | |  |
|  | AG+GG | 74 | 33 (78.5) | 41 (89.1) |  |  | |  | |  |
|  | **Genotypes** | **N** | **Asthma controlled (n, %)** | | **PR (95% CI)** | ***p*-value** | |  | |  |
|  |  |  | **ACT score≤19** | **ACT score>19** |  |  |  |  |  |  |
|  | AA | 15 | 2 (10.5) | 13 (14.6) |  | 0.728 | | Ref. | |  |
|  | AG | 57 | 9 (47.3) | 48 (53.9) |  |  | | 0.814 | | Ref. |
|  | GG | 36 | 8 (42.1) | 28 (31.4) |  |  | | 0.466 | | 0.434 |
|  | G allele | 129 | 25 (65.7) | 104 (58.4) | 1.12 (0.86-1.46) | 0.401 | |  | |  |
|  | A allele | 87 | 13 (34.2) | 74 (41.5) |  |  | |  | |  |
| **Dominant pattern** | AG+AA | 72 | 11 (57.9) | 61 (68.5) | 0.84 (0.56-1.27) | 0.372 | |  | |  |
|  | GG | 36 | 8 (42.1) | 28 (31.4) |  |  | |  | |  |
| **Recessive pattern** | AA | 15 | 2 (10.5) | 13 (14.6) | 0.72 (0.18-2.93) | 0.641 | |  | |  |
|  | AG+GG | 93 | 17 (89.4) | 76 (85.3) |  |  | |  | |  |
|  | **Genotypes** | **N** | **Bronchodilator reversibility (n, %)** | | **PR (95% CI)** | ***p*-value** | |  | |  |
|  |  |  | **FEV_1_ increase of <12% and <200 mL from baseline** | **FEV_1_ increase of ≥12% and ≥200 mL from baseline** |  |  |  |  |  |  |
|  | AA | 14 | 3 (15.0) | 11 (15.9) |  | 0.941 | | Ref. | |  |
|  | AG | 43 | 9 (45.0) | 34 (49.2) |  |  | | 0.968 | | Ref. |
|  | GG | 32 | 8 (40.0) | 24 (34.7) |  |  | | 0.793 | | 0.677 |
|  | G allele | 107 | 25 (62.5) | 82 (59.4) | 1.05 (0.79-1.38) | 0.726 | |  | |  |
|  | A allele | 71 | 15 (37.5) | 56 (40.5) |  |  | |  | |  |
| **Dominant pattern** | AG+AA | 57 | 12 (60.0) | 45 (65.2) | 0.92 (0.62-1.37) | 0.668 | |  | |  |
|  | GG | 32 | 8 (40.0) | 24 (34.7) |  |  | |  | |  |
| **Recessive pattern** | AA | 14 | 3 (15.0) | 11 (15.9) | 0.94 (0.29-3.04) | 0.918 | |  | |  |
|  | AG+GG | 75 | 17 (85.0) | 58 (84.0) |  |  | |  | |  |
|  | **Genotypes** | **N** | **Fixed airflow obstruction (n, %)** | | **PR (95% CI)** | ***p*-value** |  | |  | |
|  |  |  | **Post-BD FEV_1_<70%** | **Post-BD FEV1≥70%** |  |  |  |  |  |  |
|  | AA | 20 | 12 (17.9) | 8 (13.7) |  | 0.401 | | Ref. | |  |
|  | AG | 63 | 36 (53.7) | 27 (46.5) |  |  | | 0.821 | | Ref. |
|  | GG | 42 | 19 (28.3) | 23 (39.6) |  |  | | 0.277 | | 0.231 |
|  | G allele | 147 | 74 (55.2) | 73 (62.9) | 0.87 (0.71-1.07) | 0.216 | |  | |  |
|  | A allele | 103 | 60 (44.7) | 43 (37.0) |  |  | |  | |  |
| **Dominant pattern** | AG+AA | 83 | 48 (71.6) | 35 (60.3) | 1.18 (0.92-1.53) | 0.182 | |  | |  |
|  | GG | 42 | 19 (28.3) | 23 (39.6) |  |  | |  | |  |
| **Recessive pattern** | AA | 20 | 12 (17.9) | 8 (13.7) | 1.29 (0.57-2.95) | 0.531 | |  | |  |
|  | AG+GG | 105 | 55 (82.0) | 50 (86.2) |  |  | |  | |  |

**S6 Table.** Associations of *IL13* rs20541 genotype with asthma phenotype in asthma patients. The Chi-square test analysis of the IL-13 rs20541 genotype showed no significant differences in the polymorphism compared to clinical outcomes in asthma patients.

*Nominal significance (*p* < 0.05), **Significance after Bonferroni correction (*p* < 1.25x10^-4^). N: Number of patients, PR: Prevalence ratio, sIgE: Specific-IgE, EOS: Eosinophils, ACT: Asthma control test, FEV1: Forced expiratory volume in one second, Pre-BD FEV1: pre-bronchodilator FEV1, Post-BD FEV1: post-bronchodilator FEV1.
